# Repeated acute lower limb ischemia due to complete occlusion of an artificial vessel graft: A case report

**DOI:** 10.1002/ccr3.7276

**Published:** 2023-04-23

**Authors:** Yamato Tamura, Takehisa Abe

**Affiliations:** ^1^ Department of Cardiovascular Surgery Nara Prefectural Seiwa Medical Center Nara Japan

**Keywords:** artificial vessel graft, complication, femoral‐popliteal bypass, lower extremity ischemia, thromboembolism

## Abstract

**Key Clinical Message:**

In cases involving an occluded artificial blood vessel graft, thrombosis of the vessel can cause lower limb ischemia. When thromboembolism develops, it is essential to rule out the complete occlusion of an artificial blood vessel graft as the cause.

**Abstract:**

A 60‐year‐old woman with bilateral superficial femoral artery occlusion underwent femoral‐popliteal bypass surgery. Six months later, left vascular prosthesis occlusion occurred; 1.5 years later, an occlusive embolus developed in the deep femoral artery. The proximal prosthesis aspect was detached from the native vessel. The limb was salvaged with bypass surgery.

## 1 INTRODUCTION

The incidence of lower limb arteriosclerosis obliterans has increased with the aging of the general population. The number of patients with lower limb peripheral artery disease has also increased along with the number of patients undergoing dialysis and individuals with risk factors for atherosclerosis, such as diabetes mellitus, hypertension, and hyperlipidemia.

Although catheter treatment has been commonly used for revascularization, bypass surgery remains the primary therapeutic approach. Developments in the surgical technique and quality of artificial graft vessels have improved surgical performance. However, vessel grafts are sometimes thrombosed. When occlusions occur in the absence of complications, such as vessel graft infection, the prosthesis is left intact because removal is more invasive. In most cases, leaving the artificial vessel graft in place causes no problems. However, it can lead to occlusive thromboembolism in the native vessel, resulting in acute lower limb ischemia.

The etiology of acute lower extremity arterial occlusion has been reported to be embolism in 46%, thrombosis of an occlusive atherosclerotic lesion in 24%, multiple factors in 20%, and stent‐graft thrombosis in 10%.^1^ In this report, we discuss a rare case of acute lower limb ischemia due to complete occlusion of an artificial vessel graft.

## 2 CASE HISTORY

Our patient was a 60‐year‐old woman with risk factors for arteriosclerosis, including diabetes mellitus, hypertension, and dyslipidemia. In 2009, she underwent bilateral femoral‐popliteal bypass surgery at another hospital after being diagnosed with bilateral superficial femoral artery occlusion. During her 6‐month follow‐up visit, we observed that the synthetic vessel graft in her left leg was occluded. Since collateral circulation from the deep femoral artery developed in the interim, she underwent further follow‐up. In February 2011, approximately 1.5 years after the synthetic vessel graft occlusion in her left thigh was detected, she noticed sudden‐onset left thigh pain and was admitted to our hospital. Occlusion of the left superficial and deep femoral arteries was observed in lower extremity arteriography. The patient was diagnosed with ischemia due to deep femoral artery obstruction and underwent endovascular intervention, following which her symptoms improved. She received anticoagulation therapy to prevent distal microemboli and was discharged.

## 3 DIFFERENTIAL DIAGNOSIS, INVESTIGATIONS, AND TREATMENT

**FIGURE 1:**
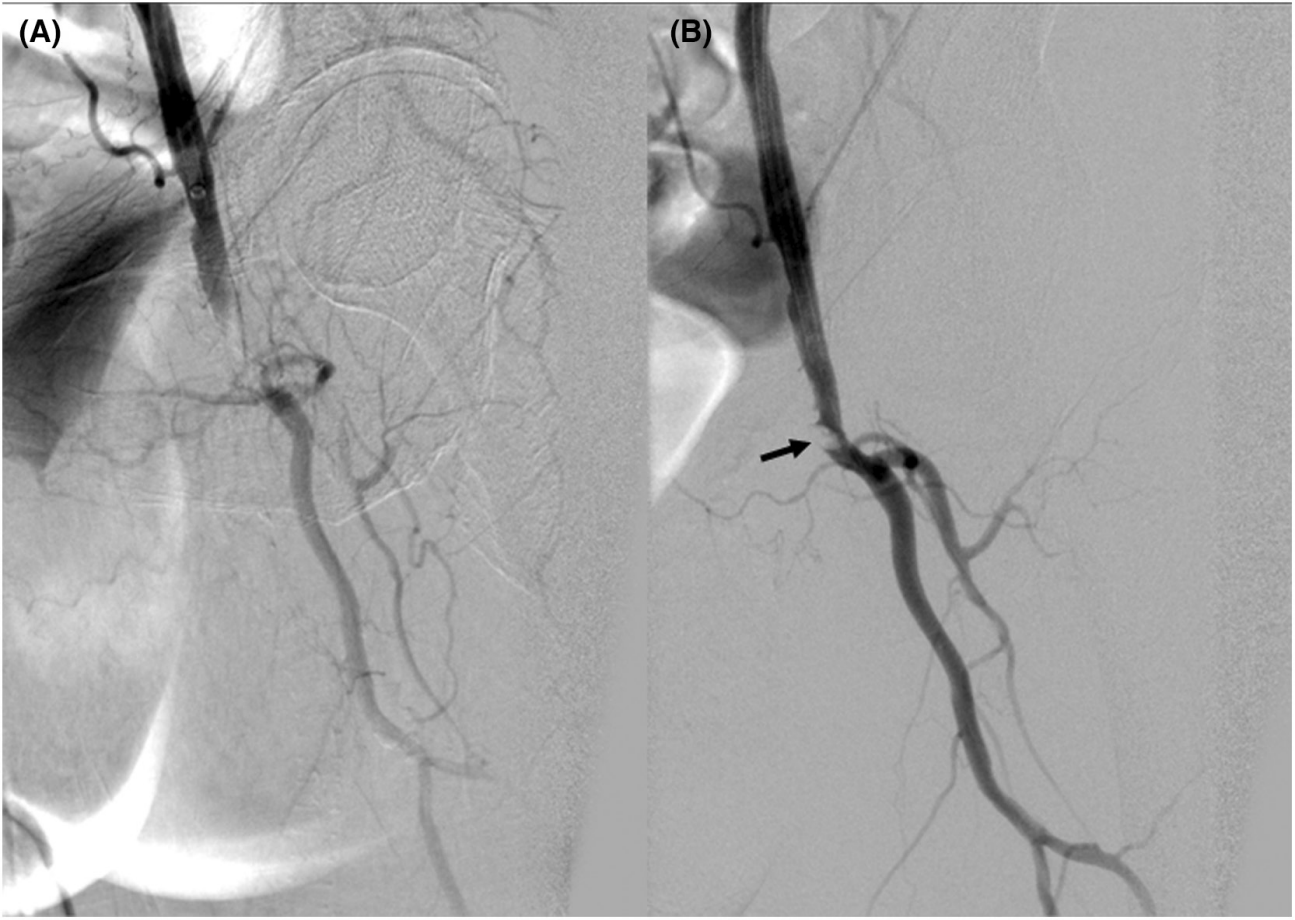
Lower extremity arteriography after catheterization reveals minimal blood flow through the left deep femoral artery and stenosis (arrow). (a) Before catheterization. (b) After catheterization.

In November 2011, the patient experienced left thigh pain and was admitted to our hospital. Poor color tone and necrosis of the left toes were noted. This improved after receiving an infusion of prostaglandin E1 (PGE1) and argatroban. Lower extremity arteriography was performed on the fifth day of admission. Left common femoral artery occlusion was observed, and endovascular treatment was performed at the same site (Figure 1). Although blood flow was noted in the deep femoral artery, common femoral artery stenosis persisted, and the patient was referred to our department.

**FIGURE 2:**
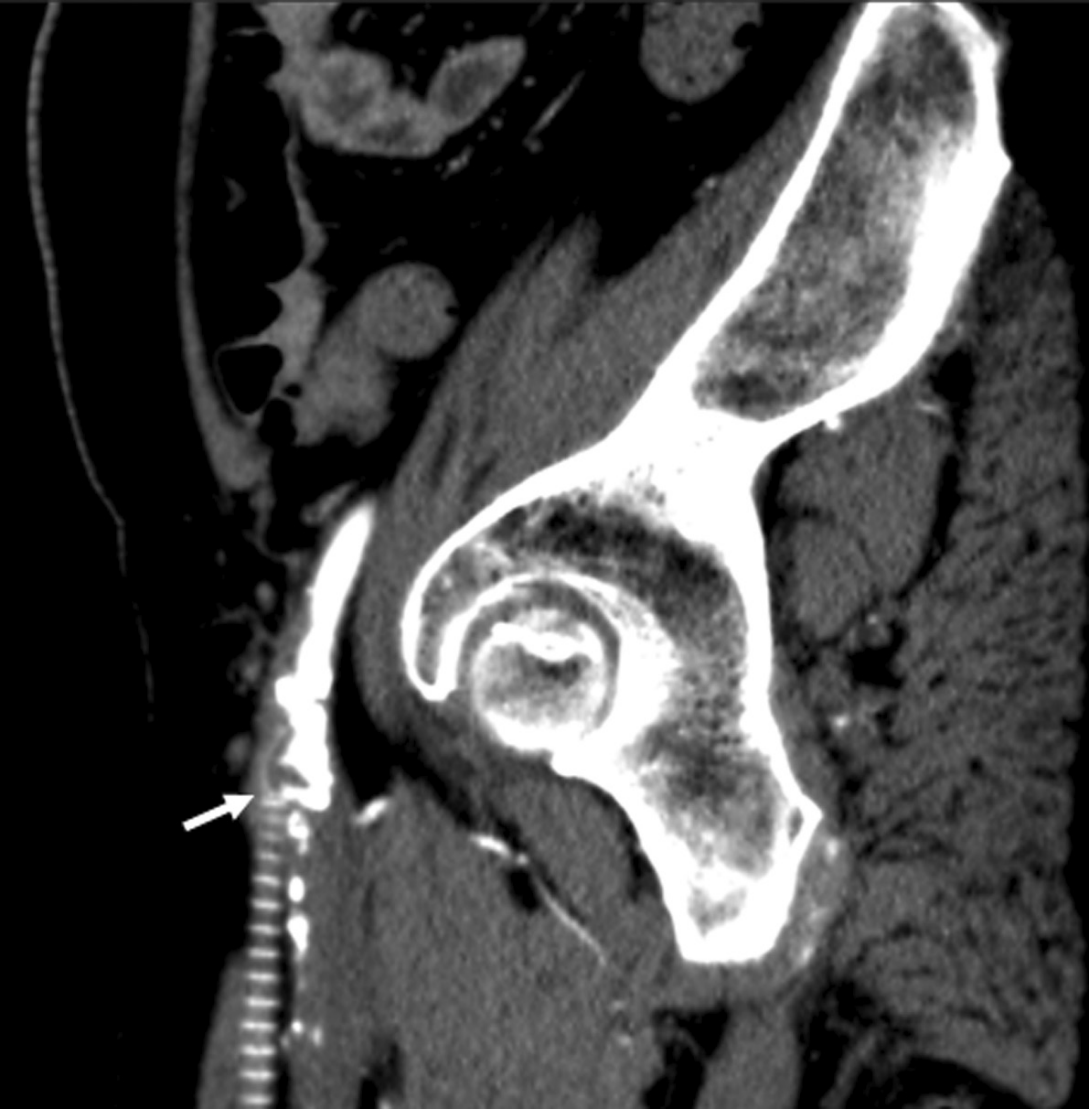
Preoperative computed tomography reveals projection of artificial ePTFE graft vessel thrombosis into the common femoral artery lumen (arrow). ePTFE, expanded polytetrafluoroethylene.

A thrombus, protruding into the lumen of the synthetic vessel graft, and lower limb emboli were detected on computed tomography (CT) (Figure 2). The patient was diagnosed with embolism and deep femoral artery occlusion due to vessel prosthesis thrombosis.

Since it is not possible to improve ischemia in the toes by only dissecting the synthetic vessel graft and attaining blood flow in the deep femoral artery, we elected to bypass the lower limb artery. After detaching the proximal end of the vessel graft, the clot was removed, and the patient underwent bypass surgery from the femoral artery to the popliteal artery below the knee using the great saphenous vein. While removing the vessel prosthesis, a dark red blood clot projecting into the common femoral artery from the blood vessel graft was noted. Stenosis was not observed in the anastomosis, and the intimal thickening was mild. The clot was removed from the deep femoral artery, and the great saphenous vein was attached to the site where the synthetic expanded polytetrafluoroethylene (ePTFE) blood vessel graft was disconnected. The great saphenous vein was endoscopically harvested, reversed, and used. This was connected distally to the popliteal artery below the knee.

## 4 OUTCOME AND FOLLOW‐UP

**FIGURE 3:**
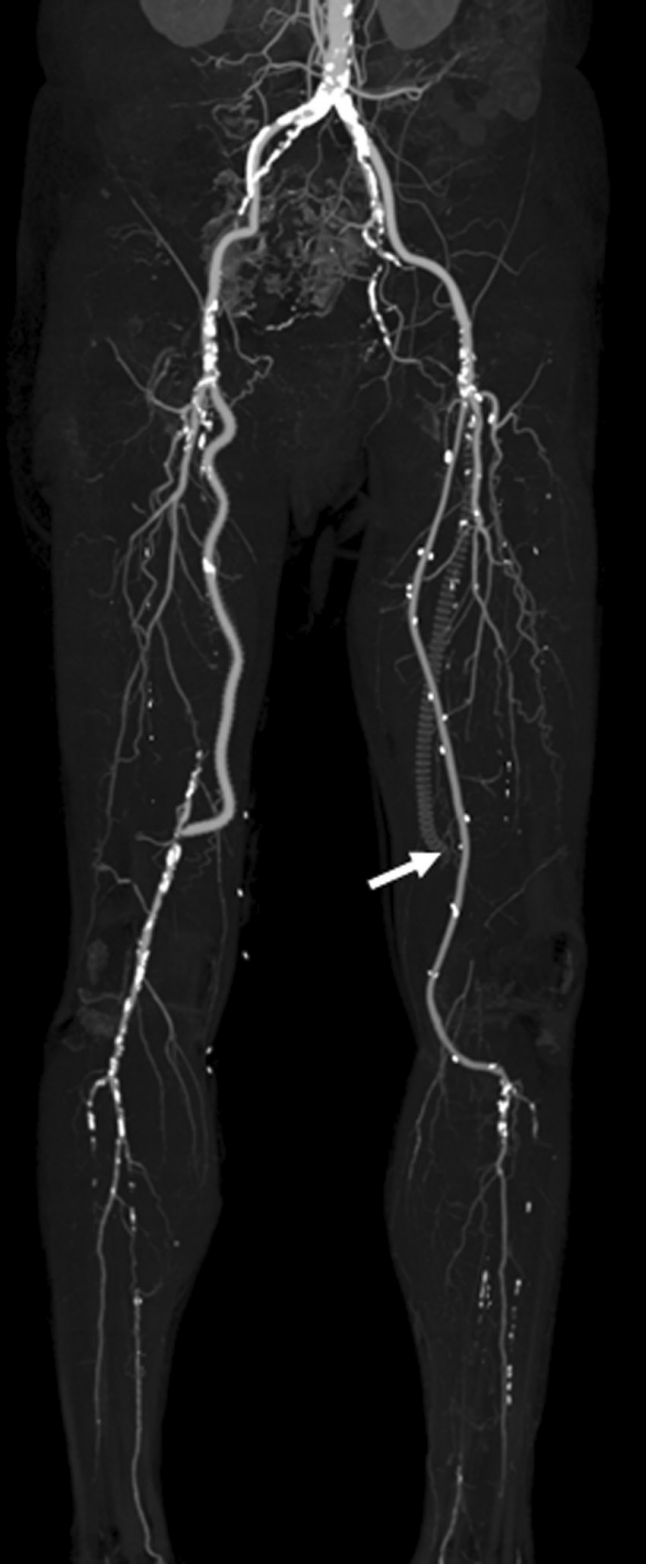
Postoperative computed tomography reveals patency of the bypass graft (arrow) with no evidence of embolism.

After the surgery, the distal extremity pulses were satisfactory. CT revealed adequate blood flow in the bypass graft, and no embolization was observed (Figure 3). The ankle–brachial index was 0.90, and left toe necrosis had resolved. No additional embolism has been reported 10 years after the surgery.

## 5 DISCUSSION

Bypass surgery is one of the primary treatments used to ensure revascularization in patients with arteriosclerosis obliterans. Although bypass improves the quality of artificial blood vessel grafts, occlusions have been reported. When occlusions occur in the absence of complications, such as graft vessel infection, the blood vessel graft is considered harmless and is left intact because removal is more invasive. However, thrombus formation in an artificial vessel graft can cause thromboembolism distal to the anastomosis.

Artificial vessel graft thrombosis has reportedly caused late‐onset embolism in the setting of axillo‐femoral bypass.^2^, ^3^, ^4^, ^5^, ^6^, ^7^ In most cases, the embolism is distal to the axillary artery, which is the source of blood flow. In special circumstances, embolism occurs in the lower limb arteries.^7^

Artificial vessel grafts used in reported cases of axillo‐bifemoral bypass include those made of Dacron and ePTFE grafts. However, thromboembolism can develop regardless of the type of vessel prosthesis. In this case, ePTFE was used.

With respect to the cause of embolism, Hartman et al. suggested that the axillary artery is driven downward by an occluded graft,^2^ and that the anastomosis deforms into a Y‐shape. Other causes identified by Khalil et al. include severe stenosis in the axillary artery anastomosis and thrombosis arising within the same site.^3^ In the present case, no native vessel deformation or apparent stenosis in the anastomosis was observed, which made it difficult to identify the cause of the embolism. We considered the danger of thromboembolism distal to the interface between the patent native vessel and the occluded synthetic ePTFE graft vessel.

In terms of treatment in the presence of an occluded vessel prosthesis, McLafferty et al. and Mawatari et al. recommend detaching the occluded artificial vessel graft from the native vessel because of the high risk of embolism.^5^, ^6^ Recurrent thromboembolism cases have been reported after performing thrombectomy only, highlighting the need to remove the vessel graft. In the present case, endovascular treatment was also performed. The synthetic ePTFE vessel graft was detached from the native circulation due to the possibility of thromboembolism recurrence. While some reports have reported favorable outcomes for cases involving an embolism distal to the axillary artery treated using covered stents, the long‐term outcomes of covered stents for the common femoral artery remain uncertain, and surgery is considered the first‐line treatment for the common femoral artery.

Given that few cases have been reported, the tendency of thromboembolism to develop at the proximal or distal end of the vessel prosthesis is unknown. In most reported cases, including the present case, the embolus formed in the proximal aspect of the vessel. Leaving the distal anastomosis of the ePTFE vessel graft connected does not eliminate the possibility of the embolism moving forward. Therefore, a strict follow‐up is necessary. In our case, the patient has not experienced embolism recurrence even 10 years after surgery.

In the present case, we observed embolism recurrence. An initially patent deep femoral artery became occluded due to artificial graft vasculature thrombosis after femoral‐popliteal bypass surgery for superficial femoral artery occlusion. The cause of thromboembolism can be eliminated by disconnecting the ePTFE vessel graft to prevent the recurrence of deep femoral artery occlusion and distal vasculature embolism. Due to the subsequent toe necrosis, revascularization was performed using the great saphenous vein. As a result, the necrosis was alleviated, and the embolism did not recur.

The current case highlights that, in cases involving an occluded ePTFE blood vessel graft, thrombosis of the vessel can cause lower limb ischemia. When thromboembolism develops, it is essential to rule out the artificial blood vessel graft as the cause since treatment methods such as endovascular intervention, thrombectomy, and anticoagulation do not prevent recurrence in these cases.

In recent years, endovascular intervention for lower limb ischemia has often been the first choice of treatment.^8^, ^9^ However, we believe it is important to recognize that when the cause is thromboembolism due to the occlusion of an artificial vessel graft, the graft should be disconnected and reported.

## 6 CONCLUSION

The artificial graft vessel may become occluded after femoral‐popliteal bypass surgery for superficial femoral artery occlusion. We discussed a recurring case of acute lower limb ischemia possibly caused by synthetic ePTFE vessel graft thrombosis 1.5 years after the operation.

In cases of unexplained acute lower limb ischemia, occlusive artificial vessel graft thrombosis can be considered. In these cases, the management plan should involve endovascular intervention, thrombectomy, and anticoagulation, as well as detachment of the occluded prosthesis from the native vessel.

## AUTHOR CONTRIBUTIONS

**Yamato Tamura:** Data curation; methodology; writing – original draft; writing – review and editing. **Takehisa Abe:** Writing – original draft; writing – review and editing.

## FUNDING INFORMATION

No funding was received for this study.

## CONFLICT OF INTEREST STATEMENT

All authors have no relevant or other potential conflicts of interest.

## ETHICAL APPROVAL

Clinicians at this medical center are not required to obtain IRB approval for case reports.

## PATIENT CONSENT

Written informed consent was obtained from the patient for the publication of this manuscript.

## Data Availability

The datasets used and/or analyzed during the current study are available from the corresponding author upon reasonable request.
